# A Comparative Study of High-Frequency Bioelectrical Impedance Analysis and Dual-Energy X-ray Absorptiometry for Estimating Body Composition

**DOI:** 10.3390/life12070994

**Published:** 2022-07-04

**Authors:** Youngseok Yi, Ji Yeon Baek, Eunju Lee, Hee-Won Jung, Il-Young Jang

**Affiliations:** Division of Geriatrics, Department of Internal Medicine, Asan Medical Center, University of Ulsan College of Medicine, Seoul 05505, Korea; yyshpm87@gmail.com (Y.Y.); eunjulee@amc.seoul.kr (E.L.); hwjung@amc.seoul.kr (H.-W.J.); dr.onezero2@amc.seoul.kr (I.-Y.J.)

**Keywords:** bioelectrical impedance analysis (BIA), dual-energy X-ray absorptiometry (DEXA), high-frequency bioelectrical impedance analysis (HF-BIA), body composition, sarcopenia, muscle mass

## Abstract

Though bioelectrical impedance analysis (BIA) is a favorable tool for assessing body composition to estimate nutritional status and physical fitness, such as sarcopenia, there are accuracy issues. Hence, high-frequency (HF) BIA equipment uses an additional frequency of 2 and 3 MHz and has been developed as a commercial model. However, there are no studies validating the accuracy and safety of HF-BIA. Therefore, this study aims to assess the validity of HF-BIA in analyzing body composition relative to dual-energy X-ray absorptiometry (DEXA). Appendicular lean mass (ALM), fat-free mass (FFM), and percentage of body fat (PBF) were assessed by HF-BIA and DEXA in 109 individuals; 50.5% (*n* = 55) were males. The average age and body mass index (BMI) were 43.4 ± 14.7 years and 25.5 ± 6.7 in males and 44.9 ± 14.1 years and 24.0 ± 6.4 in females, respectively. The HF-BIA results showed a high correlation with the DEXA results for assessing ALM (standard coefficient beta (β) ≥ 0.95), FFM (β ≥ 0.98, coefficient of determinations (R^2^) ≥ 0.95), and PBF (β ≥ 0.94, R^2^ ≥ 0.89). Body composition measured by HF-BIA demonstrated good agreement with DEXA in Korean adults.

## 1. Introduction

Sarcopenia, an age-related loss of skeletal muscle, has received a lot of attention for its physiological and clinical consequences for aging [1,2,3]. The diagnostic criteria for sarcopenia include low skeletal muscle mass with decreased muscle strength or physical performance [1,2]. Therefore, analyzing body composition and estimating skeletal muscle mass are the fundamental steps in estimating individual health [1,2,4,5]. There are a wide variety of effective devices for analyzing body composition and measuring muscle mass, including magnetic resonance imaging (MRI), computed tomography (CT), dual-energy X-ray absorptiometry (DEXA), and bioelectrical impedance analysis (BIA) [6]. Both MRI and CT are the gold standards for measuring muscle mass. However, their application in clinical settings is hindered by their high costs, low portability, and the need for highly skilled workers [7]. Therefore, DEXA is the most recommended tool for assessing muscle mass and has the advantage of being non-invasiveness, accurate, and quick, especially in diagnosing sarcopenia [1,2,3,4,5,6,8].

The two representative expert groups in sarcopenia, the European Working Group on Sarcopenia in Older People (EWGSOP) and the Asian working group for sarcopenia (AWGS), recommend both DEXA and BIA for measuring muscle mass [2]. In the guidelines of the EWGSOP, both DEXA and BIA are recognized as appropriate tools for assessing muscle mass in a usual clinical setting, and DEXA, MRI, and CT are recommended in a research setting or the specific care of patients with a high risk of adverse health outcomes [2]. Likewise, the AWGS has suggested using different cut-off values of appendicular skeletal muscle mass (ASM) in DEXA and BIA for diagnosing sarcopenia [1].

BIA is a device that quantifies impedance (the opposition to the alternating current) by analyzing a low-level electrical current passing through the body. Using specific prediction equations involving impedance values, BIA estimates intracellular water, extracellular water, total body water, fat-free mass (FFM), fat mass (FM), and percentage of body fat (PBF). BIA has the advantages of being relatively inexpensive, portable, requires minimal training, and has no risk of radiation exposure [2,6,9,10,11,12]. However, accuracy can be an issue depending on the state of body water, body mass index (BMI), and the equation of individual models in a different population [13,14,15]. Direct segmental multi-frequency (DSM) BIA has been developed to address the accuracy issues, and its effectiveness relative to that of DEXA has been validated in various clinical trials [14,15,16]. Unlike single-frequency BIA (SF-BIA), DSM-BIA analyzes the body composition of both arms and legs and the trunk separately with a wide range of multi-frequencies from 1 kHz to 1 MHz, making it possible to measure the amount of intracellular and extracellular water more precisely [10,15,16]. However, recently developed models, InBody 970 and body water analyzer (BWA) (InBody Co., Ltd., Seoul, Korea), use additional frequencies of 2 and 3 MHz and have not yet been validated in terms of accuracy and safety [17], though they are predicted to be more accurate [18].

To be recognized as an effective device for analyzing body composition, the newly developed high-frequency (HF) BIA should be validated with a comparison to DEXA, which is the standard tool for measuring muscle mass. Therefore, this study aims to validate the accuracy of HF-BIA for analyzing body composition relative to DEXA. The primary outcome measurement was appendicular lean mass (ALM) and the secondary outcome measurements were FFM and PBF.

## 2. Materials and Methods

### 2.1. Study Design

A total of 109 participants (55 men and 54 women) were enrolled via public advertisements at the Asan Medical Center, a tertiary hospital in Seoul, Korea, from October 2019 to November 2021. The inclusion criteria were adults aged 20–70 years old who could stand alone for over five minutes without assistance. Additionally, the exclusion criteria were participants who (1) had an inserted metal object such as a cardiac pacemaker, (2) had an amputated limb, (3) were unable to lie in a supine position, (4) were on their menstrual period, or (5) were pregnant.

Participants attended the geriatrics outpatient clinic at the Asan Medical Center twice for body composition assessments. For the first visit, participants were asked to refrain from eating for two hours or drinking for thirty minutes prior to testing due to the possibility of overestimating the body weight or fat mass. Participants were also asked not to perform moderate-to-intense physical activity for at least 12 h before testing and to empty their bladder before assessing body composition. In addition, all body measurements were measured at an ambient temperature. 

Upon arrival, participants had their height and weight measured. Following a ten-minute standing break, body composition was assessed twice in the same position by the InBody 970 (InBody Co., Ltd., Seoul, Korea). Then, after having a 15 min break in the supine position, BWA (InBody Co., Ltd., Seoul, Korea) measurements were conducted in the same position twice according to the electrode types (clamp-type and adhesive-type) a total of four times. In the last step, a DEXA scan was performed with the participant lying supine. Participants received consultation during the second visit based on the preceding BIA and DEXA results. In addition, the purpose and risks of the study were explained to each participant before the test, and all participants provided written informed consent. The study protocol was approved by the institutional review board of Asan Medical Center (IRB No. 2019-1401) and all procedures followed the Declaration of Helsinki.

### 2.2. BMI (Anthropometric Measurement)

BMI is a representative anthropometric measurement that provides proxies for body composition. First, each participant’s height and weight were measured using standard methods. Then, BMI was calculated by dividing the body weight (kg) by the height squared (m^2^).

### 2.3. High-Frequency BIA (HF-BIA)

Body composition was assessed using two types of HF-BIA machines, InBody 970 and BWA (clamp and adhesive type), which measured the impedance of five body segments with 1, 5, 50, 250, and 500 kHz, and 1, 2, and 3 MHz frequencies, respectively. For the InBody 970 measurement, participants were asked to stand barefoot on the floor electrodes and hold both hand electrodes. BWA measurements were taken with participants lying down and maintaining a front-facing posture with their arms away from their body and legs spread apart. The electrodes of the limbs (right arm, left arm, right leg, and left leg) were attached by a clamp or adhesive electrodes. In the BWA adhesive-type, “BWA-ES100” electrodes, which were developed by InBody Co., were used. During each measurement, all electrode sites were cleaned with an alcohol swab before the electrode placement. All measurements were repeated twice and the average of the measurements was used for the analysis.

### 2.4. Dual-Energy X-ray Absorptiometry

DEXA measures tissue absorption of high- and low-energy X-ray beams that pass through the participant in a supine position. Those measurements provide a multi-compartment assessment, including FM, FFM, and bone mineral density. The DEXA device automatically calculates the body composition of each participant. Before the scan, all metal items are removed from the participant. In the study, a Lunar Prodigy DEXA unit (GE Lunar) and the Encore Software ver.11.0 (GE Healthcare, Madison, WI, USA). was used for analysis.

### 2.5. Statistical Analysis

Data were presented as the mean ± standard deviation (SD), and a two-sided *p* < 0.05 was considered significant. Repeated measures of analysis of variance (ANOVA) were used to determine the possible differences in body composition between DEXA and HF-BIA. We conducted a linear logistic regression with a standardized beta to investigate the correlation between ALM, FFM, and PBF values measured by HF-BIA or DEXA. Between-method differences and limits of agreement (LoA) at the individual level were calculated as the mean and percentage difference (bias) between methods ± 1.96 SD using a Bland–Altman analysis. All the data were analyzed using Stata software version 17.0 (Stata Corp LP, College Station, TX, USA).

As the primary outcome was the correlation between DEXA and HF-BIA in measuring ALM, the effect size was calculated by the difference in correlation coefficient, *p* = 0.30. Hence, we determined that a sample of 109 participants would offer 90% power at an alpha level of 5% during the study protocol [19,20].

## 3. Results

Of the 109 participants of the study, 50.5% (*n* = 55) were male. The average age and BMI were 43.4 ± 14.7 years old and 25.5 ± 6.7 in males and 44.9 ± 14.1 years old and 24.0 ± 6.4 in females, respectively. Height, weight, and ALM values were significantly greater in the male participants than in the female participants. However, body fat percentage was significantly higher in females than in males (Table 1). 

**Figure 1:**
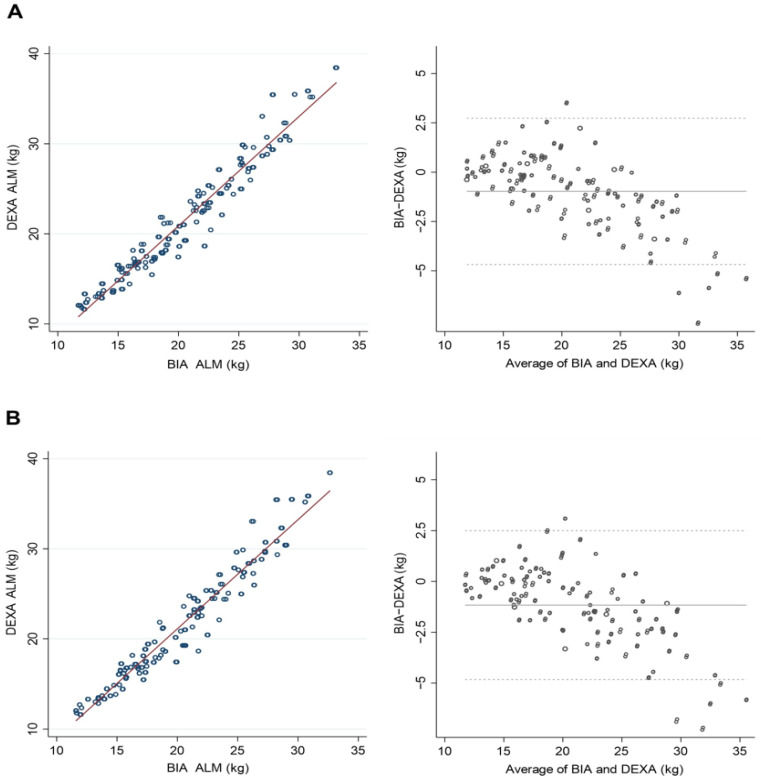
Scatterplots and Bland–Altman plots of appendicular lean mass (ALM) between dual-energy X-ray absorptiometry (DEXA) and high-frequency bioelectrical impedance analysis (HF-BIA). (**A**) Inbody970 and DEXA. (**B**) BWA (clamp) and DEXA.

**Figure 2:**
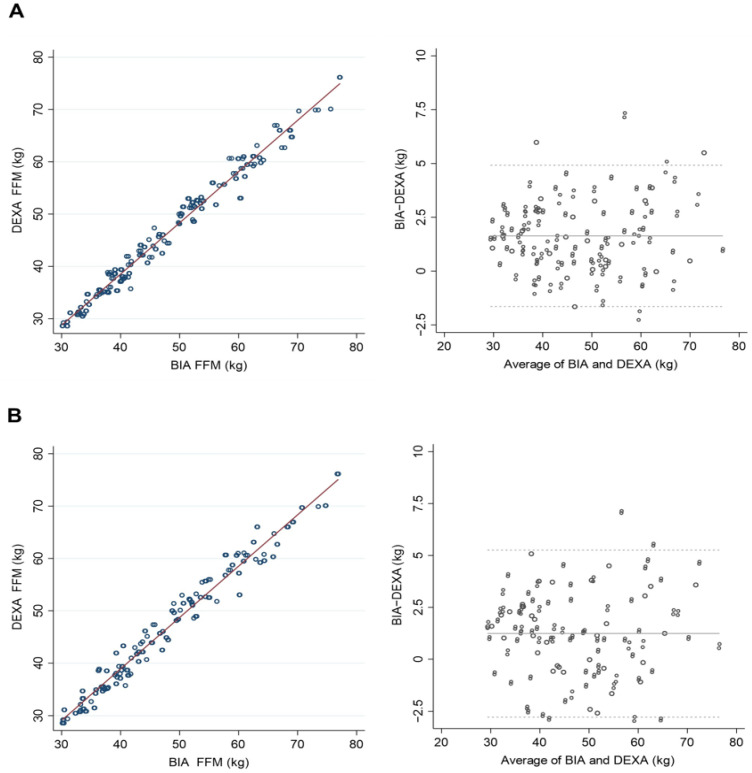
Scatterplots and Bland–Altman plots of fat-free mass (FFM) between dual-energy X-ray absorptiometry (DEXA) and high-frequency bioelectrical impedance analysis (HF-BIA). (**A**) Inbody970 and DEXA. (**B**) BWA (clamp) and DEXA.

**Figure 3:**
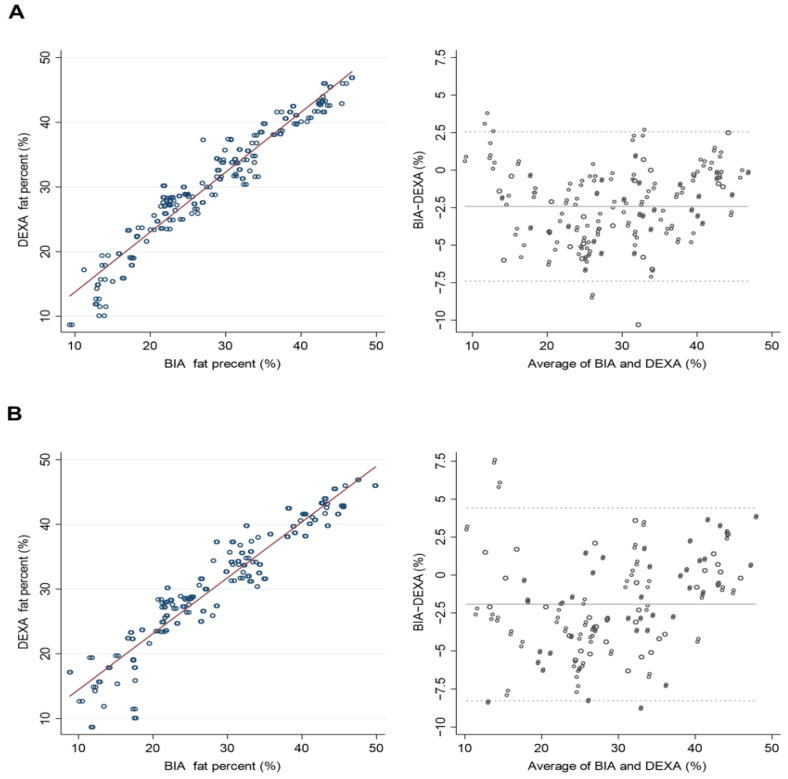
Scatterplots and Bland–Altman plots of percentage of body fat between dual-energy X-ray absorptiometry (DEXA) and high-frequency bioelectrical impedance analysis (HF-BIA). (**A**) Inbody970 and DEXA. (**B**) BWA (clamp) and DEXA.

The HF-BIA underestimated ALM and PBF and overestimated FFM compared with DEXA in both men and women (*p* < 0.01) (Table 2). A significant correlation existed between DEXA and HF-BIA measurements for estimating the amount of ALM. The standard coefficient betas (β) were over 0.95 in all three BIAs, InBody 970, BWA (clamp), and BWA (adhesive), and the coefficient determinations (R^2^) exceeded 0.90 (Table 3). Likewise, the correlations between the DEXA and HF-BIA measurements were substantial for assessing FFM (β ≥ 0.98, R^2^ ≥ 0.95) and body fat percentage (β ≥ 0.94, R^2^ ≥ 0.89) (Table 3). Both DEXA and HF-BIA were congruent in estimating ALM, FFM, and PBF in scatterplots and Bland–Altman plot analyses (Figure 1, Figure 2 and Figure 3). In assessing ALM and PBF, some proportional biases were found in the Bland–Altman plot analysis (Figure 1 and Figure 3).

## 4. Discussion

The study’s primary outcome demonstrated that HF-BIAs (InBody 970 and BWA) had a good agreement with DEXA for estimating ALM, FFM, and PBF, especially in Korean ambulatory adults. To our knowledge, this study is the first to validate the accuracy and safety of HF-BIA relative to DEXA.

Body composition assessments, including FM and FFM, are effective for assessing the nutritional status and physical fitness of healthy individuals or those with diseases such as insulin resistance or metabolic syndrome [21,22,23]. Moreover, analyzing the skeletal muscle mass (whole-body or appendicular) is important as it can be used for diagnosing sarcopenia, which has recently been in the spotlight with the increase in the global aging population [1,2,3,4,5]. Among various methods for assessing body composition with respective pros and cons, DEXA, shown to have a high correlation with MRI, has been the gold standard for measuring muscle mass [24,25,26,27]. However, BIA has been favored because of its easy application and cost-effectiveness relative to more sophisticated imaging techniques such as CT and MRI [6].

The SF-BIA prototype had measurement errors depending on the state of body water and BMI [13,14]. Moreover, the equation, including the empirical variables applied to solve these accuracy problems, had no choice but to be used only for the relevant population group. Therefore, multi-frequency BIA (MF-BIA), was later developed into DSM-BIA and made it possible to measure each body part separately and more precisely [10,15,16]. Furthermore, MF-BIA more accurately analyzes body composition without relying on empirical estimations by more accurately analyzing intracellular fluid impedance [16]. MF-BIA has overcome several accuracy issues with the state of body water and the resulting correction equation.

However, until now, the measured values by MF-BIA were heterogeneous in accuracy compared with DEXA or MRI and showed differences in the diagnosis rate of sarcopenia in various studies [28,29,30,31,32,33,34,35,36,37,38]. Nine studies evaluated the correlation between MF-BIA and DEXA for assessing muscle mass [30,32,33,34,36,37,38,39,40]. These previous studies showed a little heterogeneousness in the results of the correlation coefficient (r) and R^2^ depending on the measured portion: lean body mass (LBM, r = 0.92–0.969, R^2^ = 0.85–0.947) and ALM (r = 0.73–0.940, R^2^ = 0.880–0.917). Fang et al. concluded that the body composition of people aged 65 years and older as estimated by MF-BIA was highly correlated with that estimated by DEXA (muscle mass, r = 0.969, *p* < 0.001) [30]. Similarly, Jeon et al. found a very strong correlation between MF-BIA and DEXA for estimating ASM (R^2^ = 0.914–0.917) [38]. Although the results of R^2^ were under 0.90, Meier et al. showed that the correlation between DSM-BIA and DEXA for ALM was 0.86 when adjusted for age and sex [32]. 

There are five studies assessing the accuracy of MF-BIA relative to DEXA (ICC = 0.7–0.96, concordance correlation coefficient (CCC) = 0.4, k = 0.2–0.48 in LBM or FFM, ICC = 0.83–0.93, k = 0.397 in ALM, and ICC = 0.69–0.883 in trunk muscle mass) [28,31,36,38,39]. These prior studies showed a high correlation in assessing muscle mass between BIA and DEXA. However, according to the study circumstances, they reported that BIA over- or underestimated muscle mass compared to DEXA. Meier et al. showed that BIA overestimated total lean body muscle mass (LBM) from the reference method (ALM, mean absolute percentage error = 13%) [32]. This finding corresponded with the results of Fujimoto et al. that showed not only a high correlation between MF-BIA and DEXA for estimating ASM (r = 0.88 for males, r = 0.73 for females) but also a significant overestimation of MF-BIA in reference to DEXA (ASM, 2.5 ± 0.20 kg in males and 1.5 ± 0.17 kg in females, *p* < 0.0001) [34]. Likewise, Beaudart et al. concluded that BIA overestimated muscle mass compared with DEXA (ALM by BIA and DEXA, 9.66 kg/m^2^ and 7.93 kg/m^2^ for men, 7.63 kg/m^2^ and 6.08 kg/m^2^ for women) [28].

However, Fang et al. concluded that MF-BIA tended to underestimate muscle mass and reported that the mean difference in muscle mass between BIA and DEXA was 0.42, and the confidence interval (CI) of the two standard deviations was −3.44 to 4.28 [30]. Similarly, the accuracy of MF-BIA in the assessment of whole-body lean mass in frail elderly females was validated by Kim et al. They observed a bias (DEXA minus MF-BIA) of whole-body lean mass of 2.1 kg (95% CI, 1.8 to 2.3) and of appendicular lean mass of 1.5 kg (95% CI, 1.4 to 1.7) in a Bland–Altman plot analysis [37]. In addition, the accuracy of MF-BIA was also validated by Kim et al. in a community-dwelling older population (both men and women included). They found that there was a systematic bias (DEXA minus MF-BIA) with an underestimation of whole-body lean mass of 3.17 kg and 2.73 kg (95% CI, 3.42 to 2.93 for males and 2.57 to 2.90 for females) and of appendicular lean mass of 1.48 kg and 1.59 kg (95% CI, 1.34 to 1.62 for male and 1.49 to 1.68 for female) [33]. Moreover, Ling et al. examined the accuracy of MF-BIA and they found that MF-BIA underestimated lean body mass by 1.8% compared to the reference method of DEXA [31]. The results of these previous studies suggest the need to improve BIA accuracy.

Recently, HF-BIA was proposed to improve the accuracy of BIA. It is derived from the hypothesis that more accurate body composition measurements could be substantialized by analyzing body water more precisely and separately using larger numbers and higher frequencies. Indeed, HF-BIA equipment has recently been developed as a commercial model, for example, the InBody 970 and BWA. In particular, these brand-new HF-BIA use the world’s first 3 MHz high-frequency measurement technology to increase the accuracy of body water analysis and reduce impedance measurement errors depending on the measurement posture or external environment [17,18]. HF-BIA was proven to have accuracy and repeatability in measuring body composition in 2020, with identical examination costs compared with existing MF-BIA, except for the additional price of the electrode board for the higher frequency (2 MHz, 3 MHz). However, the validity of these machines is based on in vitro tests, whereas clinical trials in an in vivo setting have not yet been completed [13,18].

In this study, HF-BIA showed good agreement with DEXA for estimating ALM, FFM, and PBF. Furthermore, the study proved a significant correlation between DEXA and HF-BIA in ALM (β ≥ 0.95, R^2^ ≥ 0.90), FFM (β ≥ 0.98, R^2^ ≥ 0.95), and PBF (β ≥ 0.94, R^2^ ≥ 0.89). However, HF-BIA underestimated ALM and PBF and overestimated FFM relative to DEXA regardless of sex. As both DEXA and HF-BIA measure the body composition indirectly, different estimates could be derived from different devices, calibration methods, or configurations of regions of interest [33,41]. In addition, there was a proportional bias in the Bland–Altman plot analysis of the ALM. The estimates of ALM from HF-BIA tended to be underestimated in individuals with high ALM and overestimated in individuals with low ALM. This could be affected by an individual’s BMI and adiposity [31,42]. As persons with higher ALM are more likely to have higher adiposity and BMI, the estimates of the lean mass could be inaccurate and underestimated [31].

This study had some limitations. Our participants represented a specific population in terms of ethnicity. Since BIA does not estimate direct muscle mass, the estimates of muscle mass need to be calibrated by a prediction equation based on a different ethnic group for standardization. Also, age could affect the study results as the average age in Korea is higher than the rest of the global population [43,44]. Thus, our study results from the body composition analysis may not be applicable to other populations. An additional limitation is related to environmental factors; as BIA assessments were conducted in the outpatient clinic at a tertiary care university hospital, most participants were self-ambulatory and physically fit. Also, the average age of the participants was around 40 for both men and women. Therefore, the generalizability of the study could be limited. Lastly, an individual’s medical condition, medications, and skin temperature can affect the fluid balance and electrical transmissions and were not considered in this study. However, despite these limitations, our study has evident strength in validating the accuracy and safety of HF-BIA compared with DEXA for the first time. Future studies that compare the accuracy of estimating muscle mass in HF-BIA compared to MF-BIA would be clinically meaningful. Further, measuring muscle mass directly should be used as a reference value for evaluating the true accuracy of HF-BIA.

## 5. Conclusions

HF-BIA (InBody 970 and BWA) demonstrated good agreement with DEXA in measuring body composition in Korean ambulatory adults. Also, HF-BIA is more advantageous than DEXA in terms of convenience, safety, cost-effectiveness, and ease of follow-up. Therefore, HF-BIA and DEXA could be used to analyze body composition or diagnose sarcopenia in clinical settings, especially in Korean adults. However, HF-BIA might be a better choice if the situation requires frequent follow-up tests due to its inexpensiveness, ease of operation, and having no radiation safety issues. Further validations of HF-BIA in different populations are necessary. More research into whether HF-BIA has more accuracy than existing DSM-BIA in different study settings is warranted.

## Figures and Tables

**Table 1:** Baseline characteristics.

	Male (*n* = 55)	Female (*n* = 54)	*p*-Value
Age (y)	43.4 ± 14.7	44.9 ± 14.1	0.568
Height (cm)	171.6 ± 5.9	160.5 ± 6.1	<0.001
Weight (kg)	75.1 ± 20.4	62.1 ± 18.9	<0.001
BMI (kg/m^2^)	25.5 ± 6.7	24.0 ± 6.4	0.244
ALM (kg/m^2^) *	26.1 ± 5.6	17.8 ± 5.4	<0.001
Fat percent (%) *	26.6 ± 8.7	34.7 ± 7.8	<0.001

* ALM and body fat percentage were measured by dual-energy absorptiometry. ALM, appendicular lean mass; BMI, body mass index.

**Table 2:** Comparison between DEXA and HF-BIA.

	DEXA (Mean ± SD)	InBody970 (Mean ± SD)	BWA (Clamp) (Mean ± SD)	BWA (Adhesive) (Mean ± SD)	*p*-Value (ANOVA)
ALM					
Overall	21.40 ± 4.99	20.42 ± 3.92	20.23 ± 3.94	20.30 ± 3.90	<0.01
Male	25.38 ± 5.75	23.91 ± 4.00	23.77 ± 4.06	23.88 ± 3.97	<0.01
Female	17.34 ± 4.07	16.88 ± 3.40	16.63 ± 3.27	16.66 ± 3.20	<0.01
FFM					
Overall	46.5 ± 11.45	48.14 ± 11.56	47.75 ± 11.49	47.89 ± 11.47	<0.01
Male	50.21 ± 11.21	51.93 ± 11.38	51.66 ± 11.36	51.84 ± 11.31	<0.01
Female	42.74 ± 10.41	44.29 ± 10.40	43.76 ± 10.15	43.86 ± 10.13	<0.01
PBF					
Overall	30.63 ± 9.18	28.21 ± 9.54	28.71 ± 10.07	28.46 ± 10.26	<0.01
Male	29.18 ± 8.92	26.76 ± 9.18	27.08 ± 9.58	26.78 ± 9.79	<0.01
Female	32.11 ± 9.20	29.70 ± 9.67	30.37 ± 10.29	30.17 ± 10.44	<0.01

ALM, appendicular lean mass; ANOVA, analysis of variance; BMI, body mass index; BWA, body water analysis; DEXA, dual-energy X-ray absorptiometry; FFM, fat-free mass; HF-BIA, high-frequency bioelectrical impedance; PBF, percentage of body fat; SD, standard deviation.

**Table 3:** Correlations between parameters by DEXA and HF-BIA.

	ALM			Fat-Free Mass			Percent Body Fat		
	β	R^2^	RMSE (kg)	β	R^2^	RMSE (kg)	β	R^2^	RMSE (%)
InBody 970									
Overall	0.971	0.943	1.567	0.989	0.979	1.663	0.964	0.929	2.448
Male	0.956	0.914	1.692	0.988	0.977	1.714	0.962	0.925	2.447
Female	0.960	0.923	1.139	0.988	0.976	1.621	0.964	0.929	2.465
BWA (clamp)									
Overall	0.971	0.944	1.523	0.984	0.968	2.045	0.947	0.897	2.937
Male	0.954	0.911	1.728	0.984	0.968	2.002	0.949	0.900	2.837
Female	0.965	0.931	1.075	0.980	0.961	2.080	0.944	0.891	3.049
BWA (adhesive)									
Overall	0.969	0.939	1.587	0.985	0.970	1.982	0.953	0.908	2.773
Male	0.952	0.906	1.769	0.985	0.971	1.929	0.955	0.912	2.655
Female	0.962	0.926	1.110	0.981	0.963	2.017	0.949	0.902	2.902

DEXA, dual-energy X-ray absorptiometry; HF-BIA, high-frequency bioelectrical impedance; ALM, appendicular lean mass; B, standardized beta; BWA, body water analysis; DEXA, dual-energy X-ray absorptiometry; RMSE, root mean square error.

## Data Availability

The data presented in this study are available on request from the corresponding author.
